# Prevalence and prognostic significance of malnutrition risk in patients with tuberculous meningitis

**DOI:** 10.3389/fpubh.2024.1391821

**Published:** 2025-03-12

**Authors:** Can Guo, Ke-Wei Liu, Jing Tong, Meng-Qiu Gao

**Affiliations:** ^1^Department of Tuberculosis, The First Affiliated Hospital of Xinxiang Medical University, Weihui, Henan, China; ^2^Department of Tuberculosis, Beijing Chest Hospital, Capital Medical University/Beijing Tuberculosis and Thoracic Tumor Research Institute, Beijing, China

**Keywords:** tuberculous meningitis, malnutrition, cohort study, prevalence, prognostic

## Abstract

**Background:**

The residual risk of mortality or neurological disability is high in tuberculous meningitis (TBM), but there are not many effective treatments for TBM. Malnutrition is a modifiable risk factor for patients with tuberculous; however, the relationship between nutritional risk and neurological prognosis is not clear. In the present study, we aimed to explore the association between malnutrition risk and neurological outcome in patients with TBM.

**Methods:**

A retrospective cohort study was conducted from December 2010 to January 2021. Malnutrition risks were evaluated by nutritional scales, including controlling nutritional status score (CONUT), geriatric nutritional risk index (GNRI), and prognostic nutritional index (PNI). The primary outcome was a poor recovery measured by a modified Rankin Scale (mRS) at 1-year follow-up. Malnutrition risk was estimated, and the association between malnutrition and follow-up outcome was analyzed.

**Results:**

A total of 401 participants were analyzed in the study. According to CONUT, GNRI, and PNI, 299(74.56%), 231(57.61%), and 107(26.68%) patients were with malnutrition risk on admission. At 1-year follow-up, a total of 115 patients (28.67%) were with poor recovery. After adjustment for confounding factors, the association between moderate malnutrition (OR = 1.59, 95% CI 1.00–3.59, *p* = 0.050) and severe malnutrition (OR = 3.76, 95% CI 1.03–12.63, *p* = 0.049) was estimated by CONUT and was significantly associated with poor outcome. For each point increase in COUNT score (OR = 1.12, 95% CI 1.00–1.27, *p* = 0.059), the odds of poor functional recovery increased by 12%.

**Conclusion:**

Malnutrition in TBM patients was related to an increased risk of poor neurological recovery in the long-term follow-up. Our study stressed the importance of assessing malnutrition in TBM patients.

## Introduction

Tuberculosis (TB) affects 10 million people each year in worldwide (1), tuberculous meningitis (TBM) is the most severe extrapulmonary TB, a residual risk of mortality or neurological disability in up to 50% (2), but the current treatment strategy for TBM is limited (3, 4). Malnutrition is a modified risk factor associated with higher mortality in TB patients (5, 6). Previous studies reported malnutrition as an independent risk factor for treatment failure (7) and TB re-activation (8). The prevalence of malnutrition in TB patients was estimated to be nearly 50% (5, 6); however, nutritional status is often ignored and lack of screening in clinical settings.

The 2013 WHO recommends body mass index (BMI) as a tool for screening nutritional status in TB patients (9), which could not fully assess malnutrition (10). NRS-2002 scale has been used for evaluating malnutrition for TB patients in a previous study, but NRS-2002 fails to quantitatively measure the severity of malnutrition and also lacks objective assessment (11). Recently, several clinical scales have been used for objectively assessing malnutrition risk, including the controlling nutritional status (CONUT) score (12), geriatric nutritional risk index (GNRI) (13), and prognostic nutritional index (PNI) (14). Previous studies have demonstrated that these nutritional scales in patients with cardiovascular disease (15–17), stroke (18), and cancers (19) can be a useful indicator for predicting treatment outcomes. However, the association between malnutrition assessed by these nutritional scales and neurological outcomes in patients with TBM remains unclear.

In the present study, we hypothesized that malnutrition may increase the risk for poor outcomes in TBM patients. We aimed to explore the malnutrition identified by nutritional scales in TBM patients. Moreover, we further analyze the association between nutritional status and neurological outcome, which may provide evidence for nutritional intervention in future studies.

## Materials and methods

### Study design

This study was based on single-center, retrospective data from December 2010 to January 2021. Patients who met the recommended diagnostic criteria were eligible for this study (20). Included patients are given a diagnosis of definite, probable, or possible TBM depending on the recommended scoring scale (20). Exclusion criteria were as follows: patients with prior TB medications and immunity inhibitor before admission; with a medical history of cancer or other end-stage disease; with missing records of body height, weight, or blood index used to calculate nutritional risk. Informed consent was obtained from patients or their relatives. The study protocol was reported in accordance with the “Strengthening the Reporting of Observational Studies in Epidemiology” (STROBE) guideline (21).

### Data Collection

Demographic information, body height or weight, and medical history were collected at baseline. Diagnosis of definite TBM should fulfill the criteria: clinical symptom plus one or more of the following: acid-fast bacilli seen in the CSF; *Mycobacterium tuberculosis* cultured from the CSF; a CSF positive nucleic acid amplification test. Probable TBM is defined as clinical symptom plus a diagnostic score of 10 or more points (when brain imaging is not available) or 12 or more points (when brain imaging is available). Possible TBM is defined as clinical symptom criteria plus a diagnostic score of 6–9 points (when brain imaging is not available) or 6–11 points (when brain imaging is available). Possible TBM cannot be diagnosed without a lumbar puncture or brain imaging (10). TBM severity was staged based on the British Medical Research Council (BMRC) criteria at admission, and a clinical severity between grades I and III was given for diagnosis.

Weight divided by height square [kg/m^2^] was to calculate BMI. BMI categories were classified into underweight (<18.5 kg/m^2^), normal weight (18.5–24.9 kg/m^2^), overweight (25.0–29.9 kg/m^2^), and obesity (≥30.0 kg/m^2^). Peripheral blood index was collected from blood routine and biochemical report in the hospital. All the laboratory indexes of blood samples were obtained from the first-time results at admission.

### Nutritional screening scales and follow-up outcome

Nutritional scores, including the CONUT, GNRI, and PNI, were calculated to investigate malnutrition in TBM patients. The CONUT included three parameters (lymphocyte count, serum albumin, and cholesterol) to calculate the scores. The total scores range from 0 to 12, and malnutrition evaluated by CONUT was categorized as follows: normal (score 0–1), mild risk (score 2–4), moderate risk (score 5–8), and severe risk (score 9–12) (12).

The GNRI included three parameters (present body weight, ideal body weight, and serum albumin), and the score was calculated according to the following formula: 41.7 × present weight [kg]/ideal body weight [kg] + 1.519 × serum albumin [g/L]. Ideal body weight was calculated based on the following equation: for men, ideal body weight = height in cm − 100-([height in cm − 150]/4); and for women, ideal body weight = height in cm − 100 − ([height in cm − 150]/2). Nutritional risk evaluated by GNRI was defined as follows: normal (score > 98), mild risk (score 92–98), moderate risk (score 82–91), and severe risk (score < 82) (13).

The PNI included two parameters (lymphocyte count and serum albumin), which were calculated by the formula: 0.005 × total lymphocyte count (mm^3^) + 10 × serum albumin (g/dl). Nutritional risk evaluated by PNI was classified as follows: normal (score > 38), moderate risk (score 35–38), and severe risk (score < 35) (14).

Follow-up was performed at 1 year after admission by face-to-face or telephone interview to assess the long-term neurological functional outcome. The outcome measure was evaluated by the modified Rankin Scale (mRS) (22). The poor outcome and good outcome were defined as mRS scores of 3–6 and 0–2, respectively.

### Statistical analyses

Descriptive characteristics were reported as percentages for categorical variables or mean with standard deviation for continuous variables. The χ2 test, Fisher exact test, Student *t*-test, or the Mann–Whitney U-test was performed for statistical analysis when appropriate. The nutritional risk was measured by objective scales. The following variables were adjusted in the logistic regression model: age, gender, diagnosis classification, BMRC stage, cerebral infarction, hydrocephalus, and cerebrospinal fluid protein (*p* < 0.05 by univariate analysis or clinical confounding factor). Sensitivity analyses were performed by different adjusted models. All tests were two-tailed, and a *p*-value of <0.05 was considered statistically significant. A nomogram combined nutritional score with risk factors for predicting outcomes was developed. All tests were two-tailed, and a *p*-value of <0.05 was considered statistically significant. All analyses were conducted using R version 4.2.0.

## Results

### Baseline characteristics

There were 486 patients enrolled in the cohort at baseline (Supplementary Table 1). After excluding patients with prior TB medications, immunity inhibitor before admission (*n* = 45), with a medical history of cancer or other end-stage disease (*n* = 12), and patients with missing record of body height, weight, or blood index used to calculate nutritional risk (*n* = 28), a total of 401 patients were analyzed in the study. The mean age was 38.64 (±18.23) years, and 52.80% were men. The baseline information of study patients is shown in Table 1.

**Table 1:** Baseline information of study participants with neurological outcome.

Variables	Overall (*n* = 401)	Poor outcome (*n* = 115)	Good outcome (*n* = 286)	*p*-value
Demographics, *n* (%)				
Age, y	38.64 ± 18.23	44.86 ± 20.45	36.14 ± 16.65	<0.001
Men	212 (52.80)	60 (52.20)	152 (53.10)	0.912
Body mass index, kg/m^2^	21.63 ± 3.07	21.52 ± 3.05	21.67 ± 3.08	0.426
Hypertension	53 (13.20)	18 (15.70)	35 (12.20)	0.361
Diabetes	45 (11.20)	16 (13.90)	29 (10.10)	0.280
Pulmonary TB	265 (66.1)	79 (68.7)	186 (65.0)	0.484
Military TB	128 (31.90)	35 (30.40)	93 (32.50)	0.686
Drug-resistant TB	8 (2.0)	5 (4.3)	3 (1.0)	0.047
Anemia	110 (27.4)	40 (34.8)	70 (24.5)	0.036
Clinical features, *n* (%)				
Diagnosis classification				0.607
Probable TBM	186 (46.30)	55 (47.80)	131 (45.80)	
Possible TBM	178 (44.30)	52 (45.20)	126 (44.10)	
Definite TBM	37 (9.20)	8 (7.00)	29 (10.10)	
BMRC				<0.001
Stage I	201 (50.10)	27 (23.50)	174 (60.80)	
Stage II	148 (36.90)	55 (47.80)	93 (32.50)	
Stage III	52 (12.90)	33 (28.70)	19 (6.60)	
Onset to admission, days	27.02 ± 34.68	27.02 ± 32.64	27.68 ± 35.52	0.863
Brain CT/MRI, *n* (%)				
Tuberculoma	173 (43.10)	45 (39.10)	128 (48.80)	0.304
Meningeal enhancement	104 (25.90)	28 (24.30)	76 (26.60)	0.646
Cerebral infarction	79 (19.70)	32 (27.80)	47 (16.40)	0.009
Hydrocephalus	45 (11.20)	28 (24.30)	17 (5.90)	<0.001
Cerebrospinal Fluid, mean ± SD				
Leukocyte count,10^6^/L	204.49 ± 260.11	202.64 ± 240.62	194.22 ± 303.412	0.098
Glucose, mmol/L	2.18 ± 2.10	2.15 ± 1.42	2.18 ± 1.11	0.336
Chloride, mmol/L	112.70 ± 7.65	112.07 ± 8.37	112.95 ± 7.35	0.263
Protein, mg/dl	156.88 ± 93.20	183.41 ± 96.38	146.23 ± 89.89	<0.001
Antituberculosis therapy, *n* (%)				
Isoniazid	387 (96.5)	110 (95.7)	277 (96.9)	0.553
Rifampicin	343 (85.5)	99 (86.1)	244 (85.3)	0.842
Ethambutol	325 (81.0)	85 (73.9)	240 (83.9)	0.021
Pyrazinamide	393 (98.0)	110 (95.7)	283 (99.0)	0.047
Fluoroquinolones	121 (30.2)	39 (33.9)	82 (28.7)	0.301
Other drugs	53 (13.2)	20 (17.4)	33 (11.5)	0.118

Continuous variables are expressed as mean ± SD, and categorical variables are expressed as frequency (%). TB, tuberculosis.

### Nutritional risk assessed by clinical scales

**Figure 1:**
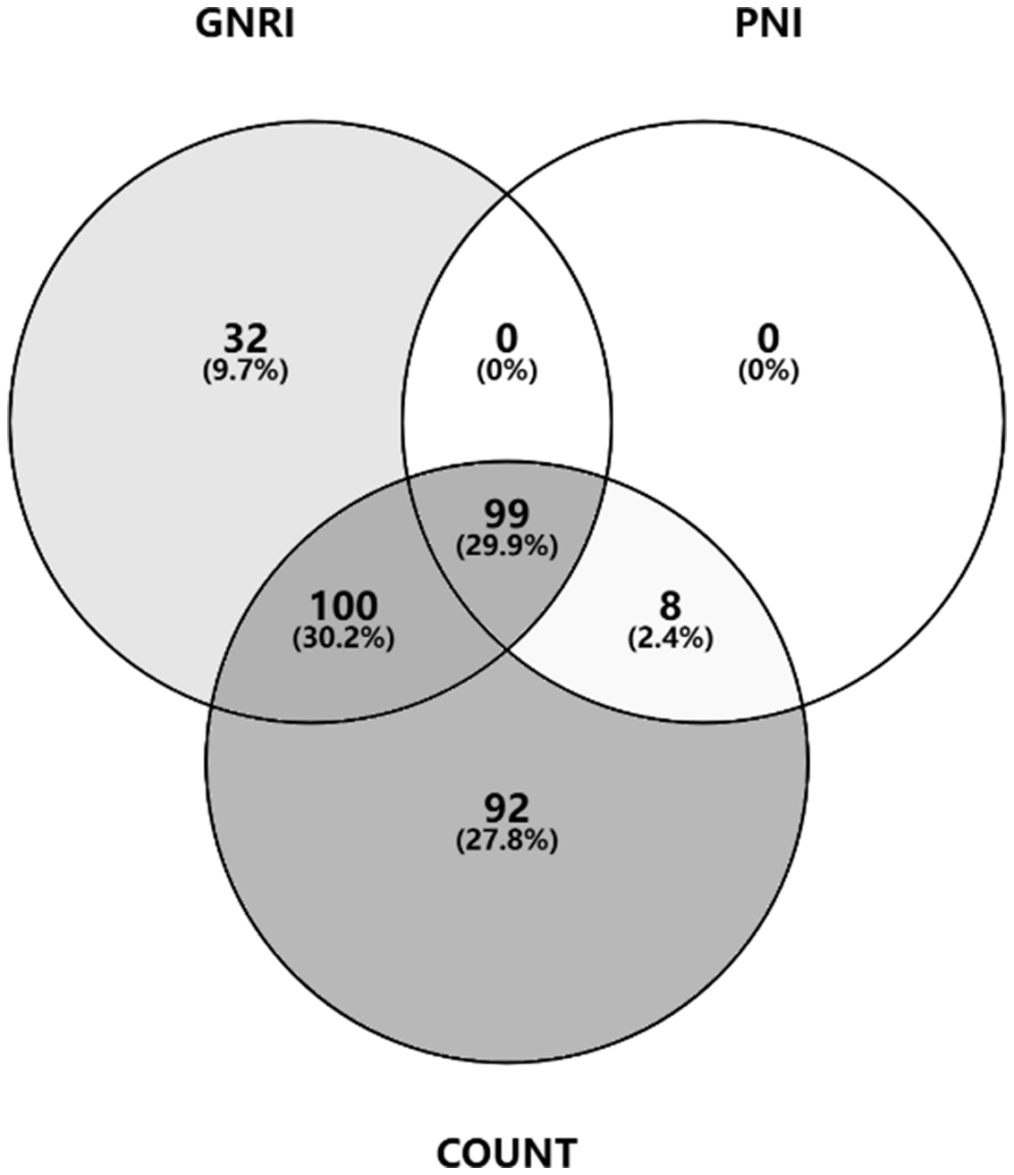
Malnutrition risk estimated by the nutritional scales.

A total of 53 (13.20%) patients were at malnutrition risk measured by the underweight BMI. According to CONUT, GNRI, and PNI, 299(74.56%), 231(57.61%), and 107(26.68%) patients were at risk of malnutrition (Table 2). The Venn diagram shows the malnutrition risk measured by the nutritional scales (Figure 1).

**Table 2:** Prevalence of the malnutrition risk identified by the nutritional scores.

	Normal, %	Malnutrition risk			
		Any	Mild	Moderate	Severe
BMI*	73.07 (68.52–77.18)	13.20 (10.25–16.89)	...	...	...
CONUT	25.44 (21.31–30.05)	74.56 (69.95–78.69)	44.14(39.24–49.16)	27.18 (22.94–31.87)	3.24 (1.81–5.62)
GNRI	42.39 (37.53–47.40)	57.61 (52.60–62.47)	23.94(19.91–28.48)	25.94 (21.78–30.57)	7.73 (5.39–10.91)
PNI	73.32 (68.65–77.53)	26.68 (22.47–31.35)	...	11.97 (9.04–15.65)	14.71 (11.47–18.65)

*Patients categorized to overweight and obesity were 13.73 (10.58–17.57) %. BMI, body mass index; CONUT, controlling nutritional status; GNRI, geriatric nutritional risk index; PNI, prognostic nutritional index.

We further performed a stratified analysis on the severity of malnutrition risk. Among these patients, 177 (41.14%) and 96 (23.94%) were at mild risk measured by CONUT and GNRI; 109 (27.18%), 104 (25.94%), and 48 (11.97%) patients were at moderate risk measured by CONUT, GNRI, and PNI; 13 (3.24%), 31 (7.73%), and 59 (14.71%) patients were at severe risk measured by CONUT, GNRI, and PNI, respectively.

### Association between malnutrition risk and neurological outcome

A total of 115 patients (28.67%) were with poor neurological functional outcomes at 12-month follow-up. Univariable analyses suggested that malnutrition was associated with poor functional outcomes. Compared to normal patients without malnutrition risk, any risk measured by COUNT, moderate-to-severe risk measured by GNRI, and severe risk measured by PNI were associated with an increased risk of poor outcome at 1-year follow-up (Table 3). In multivariable analyses, after adjustment for age, gender, drug-resistant TB, anemia, diagnosis classification, British Medical Research Council stage, cerebral infarction, hydrocephalus, cerebrospinal fluid protein, and antituberculosis therapy, the association between moderate malnutrition (OR = 1.59, 95% CI 1.00–3.59, *p* = 0.056) and severe malnutrition (OR = 3.76, 95% CI 1.03–12.63, *p* = 0.049) of CONUT and 1-year poor outcome remained significant.

**Table 3:** Multivariable analyses of nutritional scores to predict 1-year poor neurological functional outcome.

Nutritional status	Events, *N* (%)	Unadjusted		Model 1		Model 2		Model 3	
		OR (95% CI)	*p*-value	OR (95% CI)	*p*-value	OR (95% CI)	*p*-value	OR (95% CI)	*p*-value
BMI									
Continuous	...	0.98(0.92–1.06)	0.661	0.98(0.91–1.06)	0.655	1.02(0.93–1.11)	0.731	1.02(0.93–1.11)	0.736
Normal	14(26.40)	Reference		Reference		Reference		Reference	
Underweight	85(29.00)	0.88(0.45–1.70)	0.701	0.91(0.46–1.80)	0.911	0.71(0.33–1.55)	0.392	0.71(0.32–1.57)	0.403
Overweight-Obesity	16(29.10)	1.00(0.53–1.89)	0.990	1.06(0.55–2.04)	0.868	1.29(0.62–2.66)	0.495	1.32(0.63–2.76)	0.456
CONUT score									
Continuous	...	1.12(1.09–1.32)	<0.001*	1.13(1.02–1.26)	0.024*	1.11(0.98–1.25)	0.078*	1.12(1.00–1.27)	0.059*
Normal	17(16.70)	Reference		Reference		Reference		Reference	
Mild risk	54(30.50)	2.19(1.19–4.05)	0.012*	1.98(1.06–3.70)	0.033*	1.68(0.96–3.27)	**0.082**	1.76(0.88–3.51)	0.089
Moderate risk	36(33.00)	2.47(1.28–4.75)	0.007*	1.81(1.03–3.72)	0.044*	1.58(0.97–3.25)	0.066*	1.59(1.00–3.59)	0.056*
Severe risk	8(61.50)	8.00(2.33–27.45)	0.001*	4.58(1.18–17.81)	0.028*	3.34(1.04–15.07)	0.042*	3.76(1.03–12.63)	0.049*
GNRI score									
Continuous	...	0.97(0.95–0.99)	0.004*	0.98(0.96–1.01)	**0.133**	0.99(0.97–1.02)	0.721	0.99(0.97–1.02)	0.618
Normal	38(22.40)	Reference		Reference		Reference		Reference	
Mild risk	26(27.10)	1.29(0.73–2.29)	0.387	1.14(0.62–2.08)	0.673	1.23(0.64–2.37)	0.537	1.24(0.64–2.43)	0.523
Moderate risk	37(35.60)	1.92(1.12–3.29)	0.018*	1.42(0.78–2.55)	0.242	1.20(0.62–2.34)	0.584	1.30(0.66–2.55)	0.445
Severe risk	14(45.20)	2.86(1.29–6.33)	0.009*	1.71(0.70–4.15)	0.239	1.21(0.45–3.26)	0.711	1.22(0.45–3.35)	0.699
PNI score									
Continuous	...	0.93(0.90–0.96)	<0.001*	0.95(0.91–0.99)	0.008*	0.97(0.93–1.02)	0.213	0.97(0.93–1.01)	0.133
Normal	71(24.10)	Reference		Reference		Reference		Reference	
Moderate risk	15(31.30)	1.43(0.73–2.78)	0.295	1.17(0.57–2.39)	0.675	0.99(0.44–2.23)	0.990	0.97(0.42–2.24)	0.945
Severe risk	29(49.20)	3.04(1.71–5.40)	<0.001*	1.93(0.98–3.79)	0.058*	1.33(0.61–2.92)	0.476	1.43(0.64–3.19)	0.244

Model 1: adjusted for age, gender, drug-resistant TB, and anemia. Model 2: adjusted for age, gender, drug-resistant TB, and anemia + diagnosis classification + BMRC stage + cerebral infarction + hydrocephalus. Model 3: adjusted for age, gender, drug-resistant TB, and anemia + diagnosis classification + BMRC stage + CSF protein + Antituberculosis therapy. BMI, body mass index; CONUT, controlling nutritional status; GNRI, geriatric nutritional risk index; PNI, prognostic nutritional index; BMRC, British Medical Research Council; CSF, cerebrospinal fluid; *p < 0.05. The bold values represented statistically significant.

### Assessed nutritional scores for predicting neurological outcome

**Figure 2:**
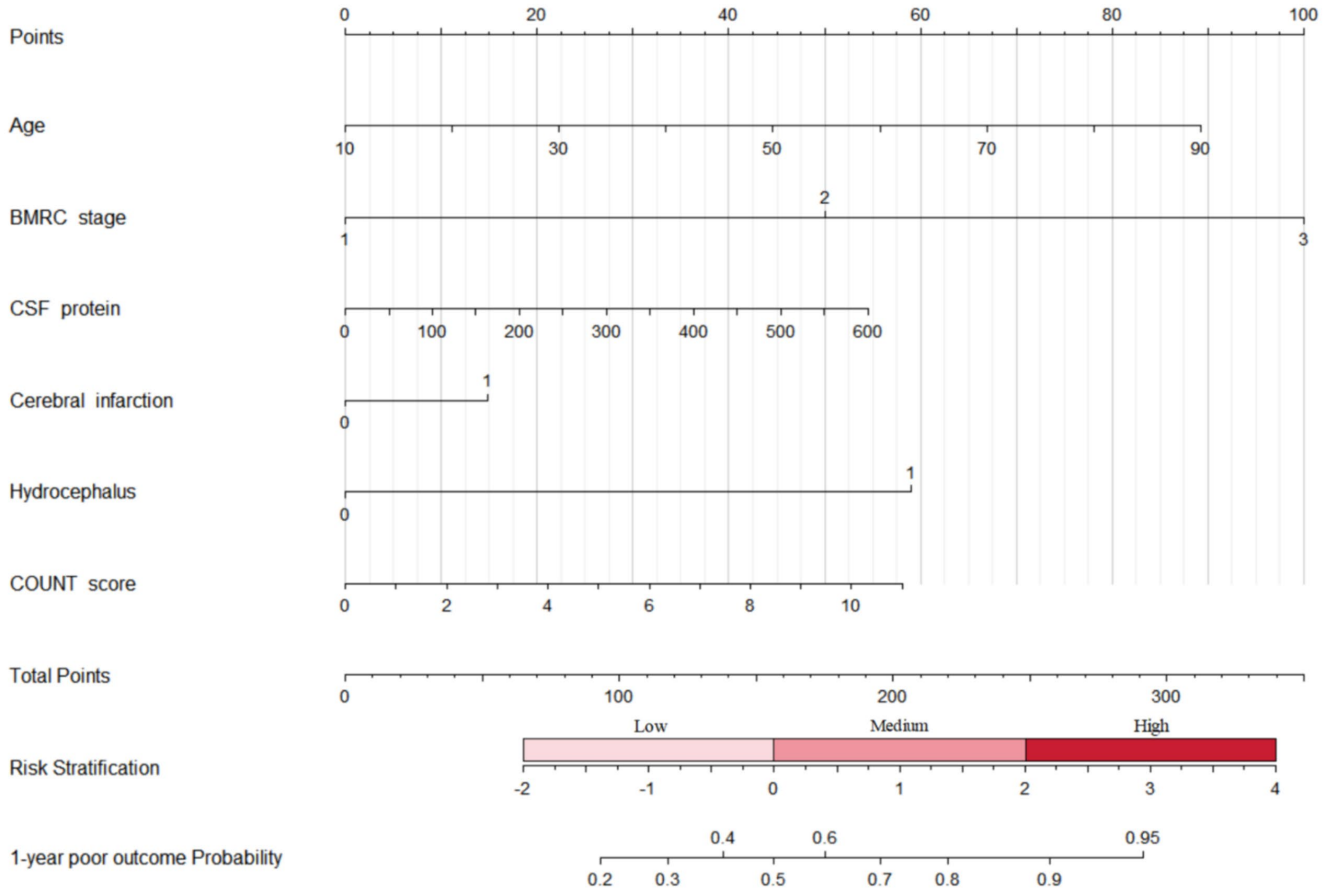
Nomogram combined CONUT score with risk factors for predicting neurological functional outcome. BMRC, British Medical Research Council; CSF, cerebrospinal fluid; CONUT, controlling nutritional status.

We treated nutritional scores as a continuous variable for sensitive analysis to predict neurological outcomes (Table 3). Univariable regression analyses suggested that nutritional scores calculated by COUNT, GNRI, and PNI were related to an increased risk of poor outcomes at 1-year follow-up. For each point increase in COUNT score (OR = 1.12, 95% CI 1.00–1.27, *p* = 0.059), the odds of poor functional recovery increased by 12%. A nomogram combined CONUT score with risk factors for predicting neurological functional outcome is shown in Figure 2.

## Discussion

To our knowledge, this is the first study to investigate the nutritional status on admission with neurological outcomes among TBM patients. The findings suggested that moderate malnutrition risk ranged from 11.97 to 27.18% and severe malnutrition risk from 3.24 to 14.71%. Moderate or severe malnutrition measured by COUNT score is associated with poor functional recovery. Combined CONUT score with risk factors can predict neurological functional outcome at 1-year follow-up.

Among TBM patients, our results showed that malnutrition risk on admission ranged between 26.68 and 74.56%, the moderate risk was between 11.97 and 27.18%, and the severe risk was between 3.24 and 14.71%. The WHO guideline recommends BMI for evaluating malnutrition in TB patients (9). However, BMI only identified 13.20% of underweight patients in our study, which underestimated the malnutrition prevalence. For patients with catabolic disorders such as TB, they may be malnourished but without a significantly changed BMI (23). This may be due to that BMI is a characteristic involved loss of weight for chronic malnutrition status, whereas most disease-associated malnutrition is a sub or acute condition, and thus does not result in significantly changed BMI in a short time (24). These findings suggested that some patients will not be identified as malnourished status when using BMI. We compared the performance of other clinical scales for evaluating nutritional status in the present study. The prevalence of malnutrition risk varied between different nutritional scales. The malnutrition risk estimated by CONUT was 74.56%, whereas 57.61% by GNRI and 26.68% by PNI, respectively. The GNRI was calculated by two parameters of weight and serum albumin (13), and the CONUT was by three parameters of serum albumin, lymphocyte count, and cholesterol level (12). Compared to CONUT, PNI only includes two parameters of albumin and lymphocyte count but without cholesterol level, which may explain the lower prevalence of malnutrition assessed by PNI (14). After adjustment for confounding factors in multivariable analyses, the results showed only CONUT was associated with poor neurological outcomes; we suggested that CONUT may be a more suitable nutritional screening tool in TBM patients.

Previous studies have reported several predictors with mortality or disability in TBM patients (25–27). A cohort study of 202 adult patients with TBM evaluated risk factors with treatment outcome and showed that hydrocephalus was the only independent risk factor for a poor outcome (25). In another cohort, prognostic factors also investigated in 154 Chinese patients with TBM revealed that limb weakness, cranial-nerve palsy, and hydrocephalus were independent predictors associated with severe disability (26). Recently, a study conducted in India indicated that radiological factors were associated with TBM outcome and suggested that using a combination of clinical and radiological prognosticate TBM (27). The prevalence of malnutrition in TB patients was high, but few studies have reported the effect of nutritional status on prognostic significance in TBM patients. In our study, we observed that moderate-to-severe malnutrition risk was significantly associated with poor neurological recovery, after adjusting for the potential risk factors. These findings stressed the importance of assessing on admission nutritional status in patients with TBM.

Our study has the following limitations. First, we only investigated on admission nutritional status in the present study. We failed to consider dynamic nutritional changes in the follow-up. Second, due to the nature of retrospective design, we used accessible parameters to investigate objective nutritional scales, but we were unable to collect the necessary variables to perform a comparison with NRS-2002 (11) and other subjective nutritional scales (28–30). Third, our dataset only included HIV-negative patients and excluded individuals whose data were incomplete for calculating malnutrition risk and outcome measures, which may lead to selection bias. Fourth, it was an observational study; despite we used multivariate analysis, the unmeasured confounding factors such as multi-vitamin deficiencies might have biased our results. Further studies with large sample sizes are needed to verify these findings.

In conclusion, moderate and severe malnutrition risk ranged between 11.97 to 27.18% and 3.24 to 14.71% in TBM patients. Moderate or severe malnutrition risk was associated with an increased risk of poor functional recovery in the long-term follow-up.

## Data Availability

The original contributions presented in the study are included in the article/Supplementary material, further inquiries can be directed to the corresponding author.
