# Supplementary material for: Prevalence and prognostic significance of malnutrition risk in patients with tuberculous meningitis
Source: Front Public Health. 2025 Mar 12;12:1391821. doi: 10.3389/fpubh.2024.1391821 (PMC11936749; doi:10.3389/fpubh.2024.1391821)
Supplement: Supplementary file 1 [file Table_1.DOCX]

**STable 1. Patients excluded and included in the present study**

| **Variables** | Overall (n=486) | Excluded (n=85) | Included (n=401) | P value |
| --- | --- | --- | --- | --- |
| **Demographics, n(%)** |  |  |  |  |
| Age, y | 38.43±18.16 | 37.41±17.88 | 38.64±18.23 | 0.559 |
| Men | 255(52.5) | 43(50.6) | 212(52.9) | 0.702 |
| Body mass index, kg/m^2^ | 21.69±3.02 | 22.01±2.81 | 21.63±3.07 | 0.267 |
| Hypertension | 66(13.6) | 13(15.3) | 53(13.2) | 0.612 |
| Diabetes | 56(11.5) | 11(12.9) | 45(11.2) | 0.652 |
| Pulmonary TB | 328(67.5) | 63(74.1) | 265(66.1) | 0.151 |
| Military TB | 160(32.9) | 32(37.6) | 128(31.9) | 0.307 |
| Drug-resistant TB | 12(2.5) | 4(4.7) | 8(2.0) | 0.143 |
| Anemia | 136(28.0) | 26(30.6) | 110(27.4) | 0.556 |
| **Clinical features, n(%)** |  |  |  |  |
| Diagnosis classification |  |  |  | <0.001 |
| Probable TBM | 271(55.8) | 85(100.0) | 186(46.4) |  |
| Possible TBM | 178(36.6) | 0(0) | 178(44.4) |  |
| Definite TBM | 37(7.6) | 0(0) | 37(9.2) |  |
| BMRC |  |  |  | 0.345 |
| Stage I | 251(51.6) | 50(58.8) | 201(50.1) |  |
| Stage II | 174(35.8) | 26(30.6) | 148(36.9) |  |
| Stage III | 61(12.6) | 9(10.6) | 52(13.0) |  |
| Onset to admission, days | 28.13±36.17 | 31.14±42.60 | 27.49±34.68 | 0.245 |
| **Brain CT/MRI, n(%)** |  |  |  |  |
| Tuberculoma | 216(44.4) | 43(50.6) | 173(43.1) | 0.210 |
| Meningeal enhancement | 135(27.8) | 31(36.5) | 104(25.9) | 0.049 |
| Cerebral infarction | 99(20.4) | 20(23.5) | 79(19.7) | 0.426 |
| Hydrocephalus | 53(10.9) | 8(9.4) | 45(11.2) | 0.627 |
| **Cerebrospinal Fluid, mean±SD** | | | | |
| Leukocyte count,10^6^/L | 201.76±253.68 | 188.91±221.86 | 204.49±260.11 | 0.669 |
| Glucose, mmol/L | 2.15±1.59 | 2.02±0.89 | 2.18±1.21 | 0.481 |
| Chloride, mmol/L | 112.96±7.58 | 114.16±7.15 | 112.70±7.65 | 0.089 |
| Protein, mg/dl | 153.18±89.61 | 135.71±67.99 | 156.88±93.21 | 0.146 |
| **Antituberculosis therapy, n(%)** | | | | |
| Isoniazid | 470(96.7) | 83(97.6) | 387(96.5) | 0.593 |
| Rifampicin | 418(86.0) | 75(88.2) | 343(85.5) | 0.515 |
| Ethambutol | 397(81.7) | 72(84.7) | 325(81.0) | 0.428 |
| Pyrazinamide | 478(98.4) | 85(100.0) | 393(98.0) | 0.189 |
| Fluoroquinolones | 157(32.3) | 36(42.4) | 121(30.2) | 0.029 |
| Other drugs | 61(12.6) | 53(13.2) | 8(9.4) | 0.336 |
